# Response of Soil Quality and Microbial Community to In Situ Return of Vegetable Residues over Three Consecutive Cropping Seasons

**DOI:** 10.3390/plants15071091

**Published:** 2026-04-02

**Authors:** Haiying Wang, Zhizhuang An, Jianbin Liu, Liang Jin, Yan Li, Yu Hu, Jie Zhu, Mingjie Yao, Xuening Xu, Dan Wei, Jianli Ding

**Affiliations:** 1 Institute of Plant Nutrition, Resources and Environment, Beijing Academy of Agriculture and Forestry Sciences, Beijing 100097, China; why2814535432@163.com (H.W.); anzz@igsnrr.ac.cn (Z.A.); liujianbin@baafs.net.cn (J.L.); jinliang19762003@aliyun.com (L.J.); huyu0805@126.com (Y.H.); yaomingjie1999@163.com (M.Y.); xvmm1316@163.com (X.X.); 2 College of Resources and Environment, Northeast Agricultural University, Harbin 150030, China; 3 Heilongjiang Academy of Black Soil Protection and Utilization, Harbin 150086, China; li.yan622@163.com; 4 College of Resources and Environment, Xinjiang Normal University, Wulumuqi 830054, China; zhujie190520@163.com; 5 College of Light Industry Science and Engineering, Tianjin University of Science and Technology, Tianjin 300457, China

**Keywords:** vegetable residues, in situ return, soil quality index, microbial community structure

## Abstract

To analyze the effects of in situ vegetable residue return on soil properties and microorganisms, this study conducted a continuous three-season in situ residue return experiment with four treatments: no return (CK), residue return (HTJ), residue return + compound microbial inoculant (HTJS), and residue return + ammonia water (HTJN). This study compared the treatment effects on soil quality. The results showed that, after the third tillage, the HTJS treatment increased soil organic carbon, total nitrogen, and mineralizable organic carbon content, and significantly enhanced the activity of soil β-glucosidase and soil peroxidase, which are related to carbon cycling enzymes compared to other treatments. There were no significant differences in bacterial or fungal α-diversity among treatments. Differences in fungal community soil β-diversity among treatments were significant. The HTJS treatment enriched organic matter-degrading bacteria *Flavisolibacter* and *Devosia*. Although HTJS increased the relative abundance of *Fusarium*, the field disease incidence index did not increase. The soil quality index (SQI), based on the minimum dataset (MDS), showed that HTJS had the highest SQI after the third tillage. Further path model analysis revealed that soil carbon components index and soil physicochemical index were the main controlling factors influencing the SQI. In conclusion, in situ residue return with a compound microbial inoculant (HTJS) is an effective strategy to simultaneously enhance soil fertility and biological activity by regulating the microbial community structure and associated enzyme activities.

## 1. Introduction

With the rapid expansion of large-scale cultivation of leafy vegetables, the production of open-field vegetables such as *Brassica rapa* L. and *Brassica oleracea* var. *capitata* L. has increased continuously, leading to a year-by-year rise in the amount of post-harvest field residues. It is estimated that the annual generation of residues from Chinese cabbage and cabbage in China has reached 122 million tons [1]. These residues are characterized by high nutrient contents such as nitrogen, phosphorus, and potassium (organic matter (OM) 520-691 g·kg^−1^, total nitrogen (TN) 27.6–37.6 g·kg^−1^, P_2_O_5_ 19.4–25 g·kg^−1^, K_2_O 32.9–54.9 g·kg^−1^), a high moisture content (typically > 80%) [2,3], and the potential to harbor pathogenic microorganisms. If incorrectly managed, these residues can create mosquito and fly breeding habitats and facilitate the spread of diseases [4]. Moreover, their nutrients are easily leached by rainfall, resulting in environmental pollution [5]. Compared with composting, which involves relatively high costs for collection and transportation, in situ incorporation into soil represents an efficient, low-cost, and simplified approach for the sustainable use of vegetable residues. In recent years, numerous studies have focused on the in situ incorporation of vegetable wastes into soil [6,7,8], but most have been conducted under protected cultivation conditions, particularly in combination with summer high-temperature greenhouse sealing techniques [6], which use elevated temperature and humidity to accelerate the decomposition of vegetable waste and suppress soil-borne pathogens. In contrast, under open-field conditions, the soil environment is influenced by multiple factors, including rainfall, temperature, and tillage practices [9,10]. Reports on continuous residue incorporation under open-field conditions are scarce. In particular, there is a lack of systematic research on the cumulative effects on soil quality, changes in microbial community structure, and the dynamics of soil-borne pathogens following residue incorporation. For the control of soil-borne diseases, biological control measures (such as the application of functional microbial inoculants) [11] and soil disinfestation methods (such as ammonia water treatment and calcium cyanamide) [12,13] are commonly employed. Microbial inoculants can significantly reduce pathogen abundance by multiple mechanisms, including direct antagonism, competition for nutrients, and induction of plant resistance [14]. Ammonia water not only serves as a nitrogen source but also provides an efficient, low-cost, and environmentally friendly agent for controlling soil-borne diseases [15]. Although previous studies have demonstrated that both microbial inoculants and ammonia water are effective in suppressing soil-borne diseases after vegetable residue incorporation [16,17], under conditions of continuous multi-season incorporation, residue accumulation, ongoing succession of soil microbial communities, and increasingly complex disease occurrence patterns raise unresolved questions. Specifically, it remains unclear whether the disease control efficacy of microbial inoculants can be sustained under continuous vegetable cropping and how soil functional properties evolve over time in relation to plant disease risk.

This study focused on post-harvest residues of open-field *Brassica rapa* L. and *Brassica oleracea* var. *capitata* L. A field-based experiment over three consecutive cropping seasons was conducted. The objectives were to (1) elucidate the effects of continuous residue incorporation on soil nutrient dynamics; (2) determine the impacts of two regulatory measures, namely, a compound microbial inoculant and ammonia solution, on soil microbial community structure; and (3) test the hypothesis that HTJS influences the soil quality index (SQI) by rapidly decomposing organic matter, thereby modifying labile carbon pools and key enzyme-mediated processes, which, in turn, alter the soil microbial community structure. Within a framework integrating multi-indicator analysis, SQI construction, and path model analysis, the relationships among soil physicochemical properties, carbon fractions, enzyme activities, microbial characteristics, and the SQI were further explored.

## 2. Results

### 2.1. Effects of In Situ Return of Vegetable Residues on Soil Physicochemical Properties

**Figure 1:**
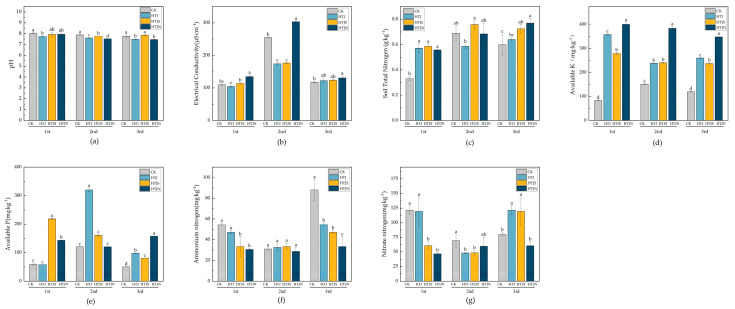
Effects of different treatments on soil physical and chemical properties; (**a**) pH, (**b**) electrical conductivity (EC), (**c**) soil total nitrogen (TN), (**d**) available potassium (AK), (**e**) available phosphorus (AP), (**f**) ammonium nitrogen (NH_4_^+^-N), (**g**) nitrate nitrogen (NO_3_^−^-N). Bars represent means ± standard error (*n* = 3). Different lowercase letters above bars indicate significant differences among treatments within the same season (one-way ANOVA followed by LSD test, *p* < 0.05). Different letters above the bars indicate significant differences among treatments (*p* < 0.05), whereas the same letters indicate no significant differences (*p* > 0.05).

The effect of continuous tillage on soil physicochemical properties is shown in Figure 1. The results indicate that all tillage treatments significantly reduced soil pH, with the HTJN treatment showing a pH decrease of 0.28 units after the third tillage compared to the CK treatment (Figure 1a). The electrical conductivity (EC) values for all treatments generally increased after the second tillage, then decreased after the third tillage, but they remained higher than the values after the first tillage. The EC value for HTJN was higher across all three tillage treatments (Figure 1b). The total nitrogen (TN) content accumulated with the number of tillage treatments, with HTJ, HTJS, and HTJN showing increases of 6.88%, 20.81%, and 28.49%, respectively, after the third tillage (Figure 1c). Tillage treatments significantly increased the available potassium (AK) content, with HTJ and HTJN showing consistently higher AK levels than CK (Figure 1d). Significant differences in available potassium (AP) content were observed among the treatments (*p* < 0.05). After the third residue incorporation, the HTJ, HTJS, and HTJN treatments were significantly higher than CK, although the HTJS treatment showed an overall downward trend (Figure 1e). After the third tillage, compared to CK, the nitrate nitrogen (NO_3_^−^-N) content in HTJ and HTJS increased by 52.07% and 49.64%, respectively, while the ammonium nitrogen (NH_4_^+^-N) content in HTJN, HTJ, and HTJS decreased by 62.01%, 37.77%, and 46.30%, respectively (Figure 1f,g).

### 2.2. Effects of In Situ Return of Vegetable Residues on Soil Carbon Fractions

**Figure 2:**
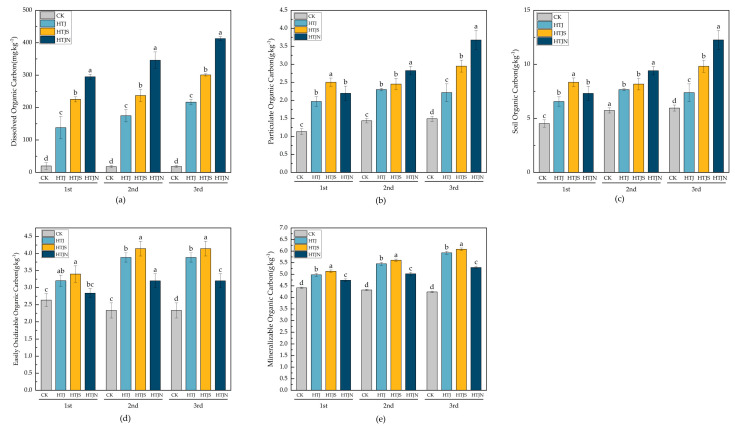
Effects of continuous in situ incorporation of three consecutive crop residues on soil carbon fractions. (**a**) Effects of vegetable residue return on dissolved organic carbon (DOC). (**b**) Effects of vegetable residue return on particulate organic carbon (POC). (**c**) Effects of vegetable residue return on soil organic carbon (SOC). (**d**) Effects of vegetable residue return on easily oxidizable organic carbon (EOOC). (**e**) Effects of vegetable residue return on mineralizable organic carbon (MOC). Bars represent means ± standard error (*n* = 3). Different lowercase letters above bars indicate significant differences among treatments within the same season (one-way ANOVA followed by LSD test, *p* < 0.05). Different letters above the bars indicate significant differences among treatments (*p* < 0.05), whereas the same letters indicate no significant differences (*p* > 0.05).

The effect of continuous tillage on soil carbon components is shown in Figure 2. Significant differences were observed between treatments (*p* < 0.05). After the third tillage, the contents of dissolved organic carbon (DOC), particulate organic carbon (POC), and soil organic carbon (SOC) followed the trend HTJN > HTJS > HTJ > CK, with HTJN being the highest (Figure 2a–c). The contents of easily oxidizable organic carbon (EOOC) and mineralizable organic carbon (MOC) (Figure 2d,e) were highest in the HTJS treatment after the third tillage, which increased by 109.8% and 43.4%, respectively, compared to CK (*p* < 0.05).

### 2.3. Effects of In Situ Return of Vegetable Residues on Soil Enzyme Activities

**Figure 3:**
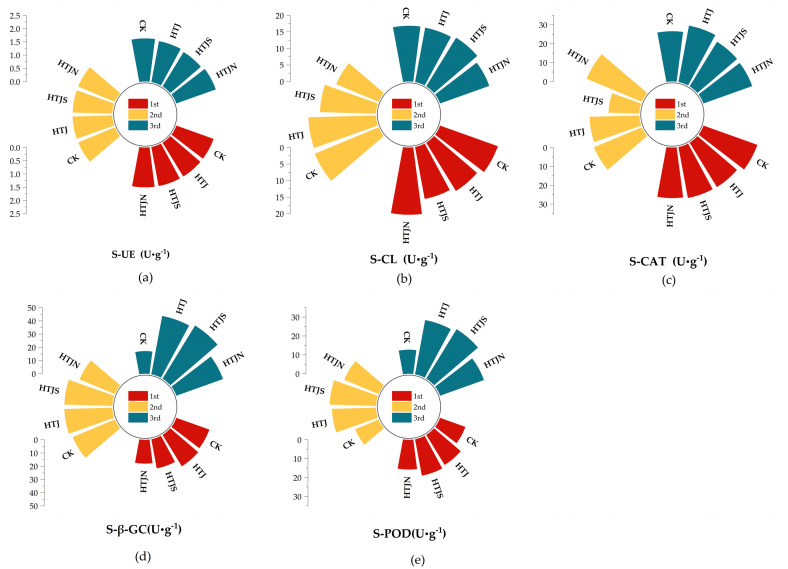
Effects of different treatments of continuous in situ incorporation of three consecutive crop residues on soil enzyme activities. (**a**) Effects of vegetable residue return on soil urease (S-UE). (**b**) Effects of vegetable residue return on soil cellulase (S-CL). (**c**) Effects of vegetable residue return on soil catalase (S-CAT). (**d**) Effects of vegetable residue return on S-β-GC. (**e**) Effects of vegetable residue return on soil peroxidase (S-POD).

The in situ incorporation of vegetable residues for three consecutive crops altered key soil enzyme activities, as shown in Figure 3, such as soil urease (S-UE) activity, which did not differ significantly among the treatments (Figure 3a). At the third residue incorporation, compared to the CK, the soil cellulase (S-CL) activity of HTJS increased by 2.59%, whereas that of HTJN decreased by 6.45% (Figure 3b). The highest soil catalase (S-CAT) activity was observed in the HTJ treatment, which increased by 14.18% compared to the CK (Figure 3c). In the HTJS treatment, soil glucosidase (S-β-GC) and soil peroxidase (S-POD) activities increased significantly, with increments of 175.22% and 141.38%, respectively, compared to CK (Figure 3d,e).

### 2.4. Effects of In Situ Return of Vegetable Residues on Soil Microbial Communities

#### 2.4.1. α-Diversity of Soil Microbial Communities

**Figure 4:**
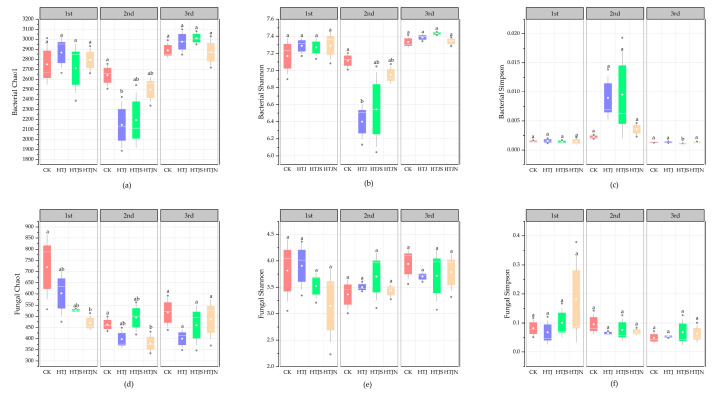
Alpha diversity indices of soil bacterial and fungal communities under different treatments of in situ return of the last crop residues for three consecutive crops. (**a**) Soil bacterial Chao1 index. (**b**) Soil bacterial Shannon index. (**c**) Soil bacterial Simpson index. (**d**) Soil fungal Chao1 index. (**e**) Soil fungal Shannon index. (**f**) Soil fungal Simpson index. Bars represent means ± standard error (*n* = 3). Different lowercase letters above bars indicate significant differences among treatments within the same season (one-way ANOVA followed by LSD test, *p* < 0.05). Different letters above the bars indicate significant differences among treatments (*p* < 0.05), whereas the same letters indicate no significant differences (*p* > 0.05).

The effects of different treatments on microbial alpha diversity in continuous tillage with three successive vegetable residue returns were assessed using the Chaol, Shannon, and Simpson indices [18] (Figure 4). In the bacterial community, there were no significant differences in the Chao1 and Shannon indices between treatments (*p* > 0.05) (Figure 4a,b). After the third tillage, these indices increased further, but no significant differences were observed between treatments. The Simpson index showed an overall decreasing trend across the three tillage events, with the HTJS treatment showing a more significant decline, although the differences between treatments were not significant (*p* > 0.05). In the fungal community, the Chao1 index for CK was significantly higher than that of other treatments after the first tillage (*p* < 0.05) (Figure 4e). After the second and third tillage treatments, there were no significant differences in the Chao1 index between treatments (*p* > 0.05). The Shannon and Simpson indices showed no significant differences between treatments (Figure 4f), indicating that the tillage treatments had no significant effect on soil microbial diversity.

#### 2.4.2. Soil Microbial Community Composition

**Figure 5:**
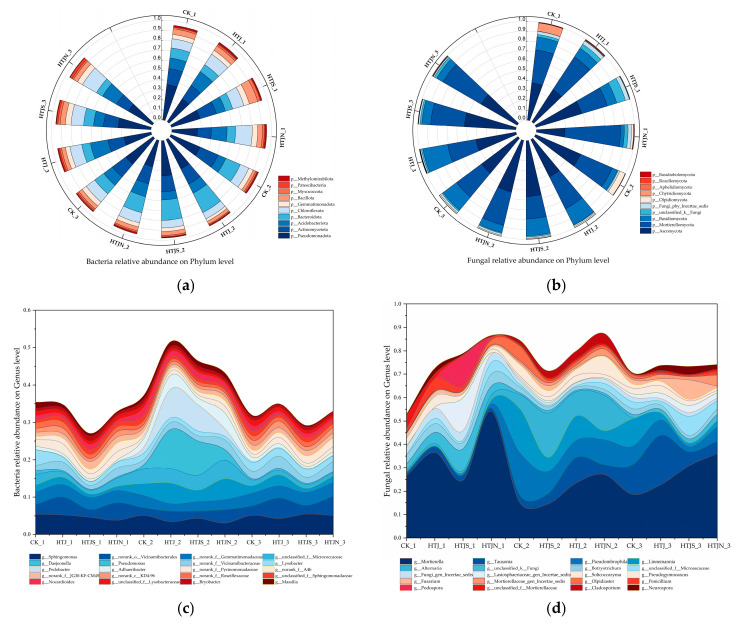
Composition and structure of soil bacterial and fungal communities. (**a**) Relative abundance of bacteria at phylum level. (**b**) Relative abundance of fungi at phylum level. (**c**) Relative abundance of bacteria at genus level. (**d**) Relative abundance of fungi at genus level.

Continuous tillage with three successive vegetable residue returns altered the soil microbial community composition. In the bacterial community, results at the phylum level (Figure 5a) showed that the dominant phyla across all treatments were Pseudomonadota (25.66–35.69%), Actinomycetota (13.73–19.62%), Acidobacteriota (8.01–17.97%), Bacteroidota (7.09–21.74%), and Chloroflexota (7.04–16.71%). After the third tillage, the relative abundance of Pseudomonadota in HTJ, HTJS, and HTJN treatments decreased by 22.7%, 2.98%, and 21.93%, respectively, compared to CK. Among the four treatments, HTJN had the highest relative abundance of Acidobacteriota and Chloroflexota, while Bacillota had the highest relative abundance in the HTJS treatment. At the genus level (Figure 5c), the dominant genera across all treatments were *Sphingomonas* (3.07–5.46%), norank_o_*Vicinamibacterales* (1.95–5.42%), and norank_f_*Gemmatimonadaceae* (1.96–3.74%). These were followed by *Daejeonella*, *Pseudomonas*, *Lysobacter*, and *Pedobacter*, which served as the sub-dominant genera.

In the fungal community, the phylum-level results (Figure 5b) showed that Ascomycota (29.04–56.35%), Mortierellomycota (22.54–56.55%), and Basidiomycota (3.85–25.66%) were the dominant phyla across all treatments. The relative abundance of Ascomycota was higher in the HTJS treatment and increased with the frequency of residue incorporation. The relative abundance of Mortierellomycota was higher in the HTJN treatment; and Basidiomycota in the HTJ treatment increased with the frequency of residue incorporation. At the genus level (Figure 5d), the dominant genera across all treatments were *Mortierella* (14.41–54.12%), *Tausonia* (0.83–21.03%), *Pseudombrophila* (0.44–18.23%), *Linnemannia* (0.82–18.91%), and *Alternaria* (0.03–18.90%). The relative abundance of *Mortierellomycota* was higher in the HTJS treatment, with an increase of 6.06% compared to the CK. *Tausonia* showed an increasing trend across all treatments, specifically increasing by 18.66% in the HTJ treatment compared to the CK. *Pseudombrophila* decreased in the HTJS treatment, while *Linnemannia* changed significantly in the CK.

#### 2.4.3. Non-Metric Multi-Dimensional Scaling (NMDS) and Differential Taxa Analysis of Soil Microbial Communities Under In Situ Vegetable Residue Return

**Figure 6:**
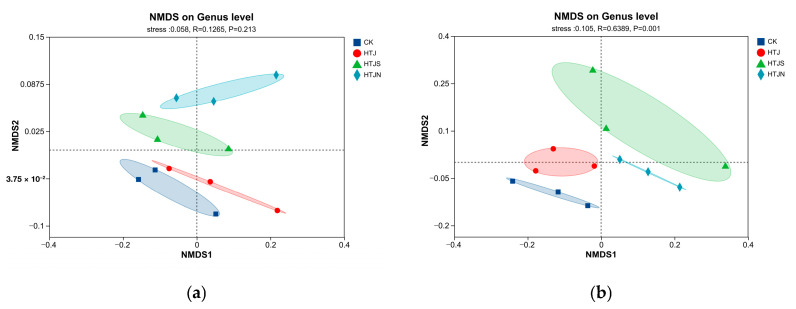

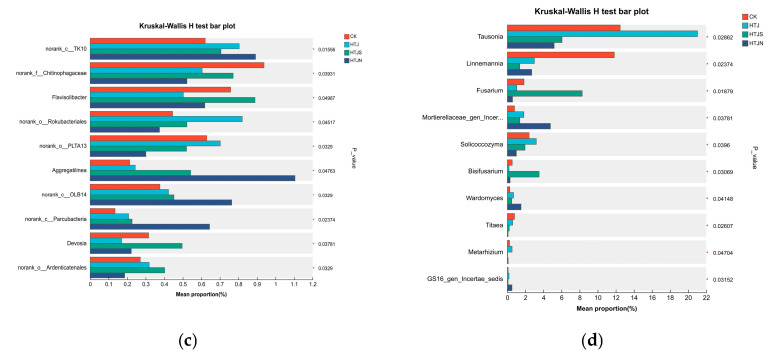
Non-metric multi-dimensional scaling (NMDS) and differential species analysis of soil microorganisms in continuous three-crop residue in situ return. (**a**) NMDS plot based on bacterial communities at the genus level. (**b**) NMDS plot based on fungal communities at the genus level. (**c**) Multiple comparative analysis of differential bacterial genera in soil. (**d**) Multiple comparative analysis of differential fungal genera in soil.

Based on NMDS analysis, the differences in soil bacterial and fungal communities among different tillage treatments are shown in Figure 6. Results showed that in the bacterial community (Figure 6a), samples from all treatments showed no distinct separation in the NMDS space. Analysis of similarities (ANOSIM) results indicated that the differences among treatments were not significant (R = 0.126, *p* = 0.213), suggesting that residue incorporation had a limited effect on the bacterial community structure. In the fungal community (Figure 6b), a distinct separation was observed between different treatments. ANOSIM showed that the fungal community structure differed significantly between treatments (R = 0.639, *p* = 0.001). NMDS analysis revealed that the HTJS and HTJN sample points were clearly separated from the CK sample points in the space, indicating that continuous tillage treatments significantly altered the fungal community structure.

Multiple comparisons of the relative abundance of differential taxa in bacterial and fungal communities were performed based on the Kruskal–Wallis test combined with Dunn’s test (*p* < 0.05). In the bacterial community (Figure 6c), *Flavisolibacter* and *Devosia* were identified as differential genera, with significantly higher relative abundances in the HTJS treatment compared to other treatments. *Aggregatilinea* and *Parcubacteria* exhibited higher relative abundances in the HTJN treatment. In the fungal community (Figure 6d), *Tausonia* and *Solicoccozyma* were significantly enriched in the HTJ treatment. Although the relative abundances of *Fusarium* and *Bisifusarium* were higher in the HTJS treatment, field disease surveys showed that the disease occurrence index of HTJS was not significantly higher than those of other treatments (details in Table S1). *Wardomyces* was higher in the HTJN treatment than in other treatments.

#### 2.4.4. Microbial Co-Occurrence Network Analysis

**Figure 7:**
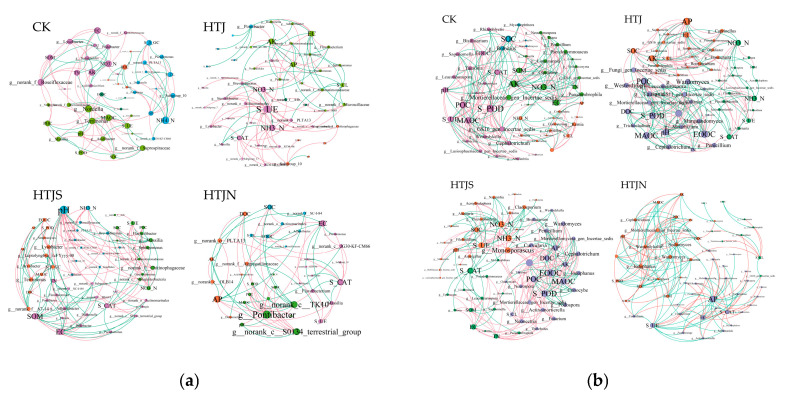
Co-occurrence networks of bacterial (**a**) and fungal (**b**) communities under different treatments.

Co-occurrence network analysis revealed differences in network topology among treatments are shown in Figure 7. Compared with CK, HTJ, HTJS, and HTJN showed higher numbers of nodes and edges in both bacterial and fungal networks, with HTJS exhibiting the greatest increase in complexity. Overall, fungal networks were more complex than bacterial networks. In HTJS, fungal nodes and edges increased by 14.29% and 63.36%, respectively, relative to the corresponding bacterial network, indicating altered microbial co-occurrence patterns under different treatments.

### 2.5. Soil Quality Index

Based on the minimum dataset (MDS) and membership, the SQI for different treatments under continuous three successive vegetable residue returns was constructed (Table 1). The cumulative SQI after three rounds of in situ vegetable residue return was as follows: HTJS (0.691) > HTJN (0.677) > HTJ (0.505) > CK (0.159). Compared to CK, all tillage treatments significantly improved the soil quality, with the HTJS treatment having the highest soil quality index.

### 2.6. Effects of Changes in Soil Indicators and Microbial Community Structure on Soil Quality

**Figure 8:**
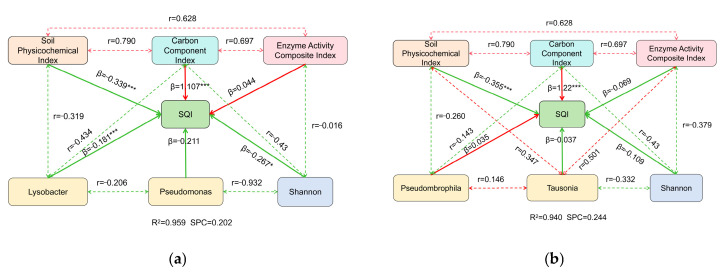
Effects of returning three consecutive harvests of vegetable residues on soil microbial community structure and diversity. (**a**) Path analysis between SQI and key bacterial genera and indicators. (**b**) Path analysis between SQI and key fungal genera and indicators. The red lines represent positive correlations, the green lines represent negative correlations, dashed lines indicate indirect effects, while solid lines indicate direct effects. The numbers on the paths are path coefficients. * *p* < 0.05, and *** *p* < 0.001.

Path analysis revealed that the main factors influencing the SQI were the soil physicochemical index, carbon component index, enzyme activity composite index, Shannon index, and key microorganisms (Figure 8). In the bacterial community model (Figure 8a), a high proportion of SQI variation was explained (R^2^ = 0.959). The carbon component index showed a significant positive direct association with the SQI (*β* = 1.107, *p* < 0.05), whereas the soil physicochemical index showed a significant negative association (*β* = −0.339, *p* < 0.05). The Shannon index displayed a weak positive association (0.044), while the remaining variables were negatively associated with the SQI. In the fungal community model (Figure 8b), the model similarly explained a substantial proportion of SQI variation (R^2^ = 0.940). The carbon component index again showed a significant positive association (*β* = 1.220, *p* < 0.05), followed by a significant negative association for the soil physicochemical index (*β* = −0.355, *p* < 0.05). The enzyme activity composite index showed a weak negative association (*β* = −0.069). *Pseudombrophila* was weakly positively associated with the SQI, whereas Tausonia was weakly negatively associated. Because some predictors were moderately correlated, standardized coefficients slightly exceeding 1 may occur in regression-based path analysis due to suppression effects. Thus, coefficients are interpreted as indicators of relative contribution rather than definitive causal effects.

## 3. Discussion

### 3.1. Effects of Three Consecutive Cycles of Vegetable Residue Return on Soil Nutrients

Soil pH is one of the important chemical properties of soil. After the third tillage, the pH in the HTJN treatment decreased by 0.28 units compared to CK. This decrease may be attributed to the organic acids produced during the mineralization and decomposition of straw residues [19,20], as well as the H^+^ generated by microbial nitrification [21]. Studies have shown that straw return significantly increases SOM, TN, and SOC [22,23]. In this study, in situ vegetable residue return similarly promoted the increase in SOC, DOC, and POC content. SOC is an important indicator for evaluating soil carbon balance and maintaining soil chemical and biochemical fertility [24]. Exogenous carbon inputs can enhance the accumulation of soil organic carbon [25]. Lin et al. found that long-term straw return significantly increased the soil SOC content [26], and the results of this study are consistent with these findings. The AP content decreased to some extent under the HTJS treatment, which could be attributed to the enhanced microbial assimilation driven by the synergistic effect of higher labile organic carbon supply and microbial inoculants. This process increased the utilization of AP during microbial growth [27,28,29], resulting in a decrease in soil AP.

### 3.2. Effects of Three Consecutive Cycles of Vegetable Residue Return on Soil Microbial Communities

Soil enzyme activities are important indicators of soil quality [30]. Wei et al. [31] reported that in situ straw incorporation increases soil microbial abundance and enzyme activities. As a rate-limiting enzyme in cellulose degradation [24], S_β_GC activity increased by more than 110% under the HTJS treatment, indicating enhanced microbial degradation of cellulose-rich organic substrates. S-POD is involved in the oxidation of recalcitrant aromatic compounds such as lignin, and its increased activity contributes to higher SOM contents [32]. In contrast, cellulase activity under the HTJN treatment decreased with an increasing incorporation frequency, likely because exogenous nitrogen inhibited certain cellulose-degrading microorganisms, thereby reducing enzyme activity [33]. This study demonstrated that continuous in situ incorporation of vegetable residues increased the richness and diversity of soil bacterial communities, which is conducive to maintaining the soil microecological balance [34], for example, the relative abundances of *Cellulomonas* and *Aspergillus* decreased by 41% and 51.81%, respectively, leading to a decline in their enzyme activities.

The HTJ treatment increased the relative abundance of *Tausonia*, a genus associated with the transformation of lignin and other refractory organic substances [35]. The HTJS treatment significantly enriched *Flavisolibacter* and *Devosia*. The efficient cellulose degradation ability of *Flavisolibacter* accelerated the decomposition and transformation of organic carbon in the vegetable residue return, increasing the soil organic carbon content and improving the soil quality [36,37]. Although an increasing trend in the relative abundance of *Fusarium* was observed in the HTJS treatment, field disease surveys revealed that the disease occurrence index did not increase as a result of the rise in potential pathogens. In the HTJN treatment, the relative abundance of Mortierellaceae gen. *Incertae sedis* (an unclassified genus within the Mortierellaceae family) and *Metarhizium* was higher. These genera are associated with organic matter decomposition and biological regulation processes [38,39]; at the same time, the HTJN treatment inhibited the abundance of beneficial bacteria such as *Sphingomonas* and the cellulase (S-CL) activity. This may be related to the drastic short-term changes in soil physicochemical properties such as pH and ionic strength following the application of ammonia water [40,41].

### 3.3. Effects of Continuous Three-Cycle Vegetable Residue Incorporation on Soil Quality Index

The SQI constructed based on MDS indicates that after the third tillage, the SQI followed the order HTJS (0.691) > HTJN (0.677) > HTJ (0.505) > CK (0.159). The MDS indicator of the SQI includes NH_4_^+^-N (a negative indicator) as well as EOOC, POC, and S-CAT (positive indicators), suggesting that different treatments may improve soil quality through mechanisms such as reducing ammonium nitrogen accumulation, enhancing active carbon components, and increasing key enzyme activities. Continuous in situ vegetable residue return significantly increased the SQI, further confirming the soil quality improvement effect of organic material return, which is consistent with the results observed by Zhang et al. in their long-term straw return study, where a continuous improvement in soil quality was noted [42]. In this study, the carbon component index was an important influencing factor of the SQI. Exogenous carbon input can promote the formation of stable carbon pools through an excitation effect [25]. The enzyme activity composite index, Shannon index, and key microorganisms had a smaller impact on the SQI. The microbial community may indirectly participate in soil biochemical processes through changes in its composition and function [43].

From the perspective of practical application, HTJ can be implemented without additional inputs, characterized by a low cost and simple operation. Although HTJS requires additional investment in microbial inoculants, it achieved the highest SQI, enabling a synergistic enhancement of economic and ecological benefits alongside soil quality improvement. In contrast, HTJN involves the application of aqueous ammonia, which entails higher costs and relatively complex procedures. Despite its SQI being comparable to that of HTJS, the use of aqueous ammonia poses greater potential environmental risks. Therefore, during the application process, factors such as input costs, soil improvement efficacy, and operability should be comprehensively evaluated to scientifically select appropriate technologies for the resource utilization of vegetable residues.

## 4. Materials and Methods

### 4.1. Overview of the Experimental Site

The field experiment was conducted at the Shenqi Seed Farm in Miyun District, Beijing, China (116°18′58.18″ E, 40°29′42.83″ N). The region is characterized as a warm temperate, semi-humid continental monsoon climate zone, with an average annual temperature of 12 °C and an average annual precipitation of 295.7 mm. The experiment was conducted using a crop rotation system of *Brassica oleracea* var. *capitata* L.—*Brassica rapa* L.—*Brassica oleracea* var. *capitata* L. *Brassica oleracea* var. *capitata* L. was sown in April 2023, with the first in situ residue incorporation conducted on 21 June. *Brassica rapa* L. was sown in September, and the second in situ residue incorporation took place on 27 November. *Brassica oleracea* var. *capitata* L. was sown again in March 2024, with the third in situ residue incorporation performed on 27 June. Before the experiment, the nutrient contents of the cultivated soil layer were as follows: pH 7.73, EC 240.00 mS/m, soil organic matter (SOM) 14.99 g·kg^−1^, total nitrogen (TN) 0.59 g·kg^−1^, available phosphorus (AP) 133.62 mg·kg^−1^, and available potassium (AK) 186.60 mg·kg^−1^, NH_4_^+^-N 0.04 mg·kg^−1^, NO_3_^−^-N 0.62 mg·kg^−1^.

### 4.2. Experimental Design and Sample Collection

The experiment adopted a randomized block design with four treatments: no vegetable residue return (CK), in situ vegetable residue return (HTJ), in situ vegetable residue return with composite microbial agents (HTJS), and in situ vegetable residue return with ammonia water treatment (HTJN). Each treatment was replicated three times, randomly distributed, with each experimental plot covering an area of 20 m^2^. The composite microbial agent used in this experiment contained Bacillus subtilis at a concentration of 1.148 × 10^9^ CFU·g^−1^ and Bacillus licheniformis at 0.25 × 10^9^ CFU·g^−1^, with an effective viable bacterial count of ≥8.0 × 10^8^ CFU·g^−1^. The application rate was 60 kg·ha^−1^, and it was uniformly applied during each straw shredding and tillage, with one application per season. Ammonia water, produced by Jinan Shunyang Chemical Technology Co., Ltd. (Jinan, China) (ammonia content 18.2%), was used at a rate of 1500 kg·ha^−1^. Vegetable residues were crushed by specialized agricultural machinery and rototilled into the soil to a depth of 20 cm. After laying drip irrigation tapes, covering with plastic mulch, and irrigation, the HTJN treatment involved the application of aqueous ammonia via drip irrigation facilities 10 days later. The residue incorporation amounts for three consecutive crops were 28.65 t·ha^−1^, 27.90 t·ha^−1^, and 29.85 t·ha^−1^, respectively. During the cultivation of *Brassica rapa* L. and *Brassica oleracea* var. *capitata* L., no base fertilizers were applied for any of the treatments, in accordance with local agricultural practices. A single application of balanced compound fertilizer (N:P_2_O_5_:K_2_O = 20:20:20, by weight) was applied during the growing season. The application rate was 300 kg/ha, providing 60 kg·ha^−1^ of nitrogen, phosphorus (as P_2_O_5_), and potassium (as K_2_O). During the growth period of *Brassica rapa* L. and *Brassica oleracea* var. *capitata* L., pest and disease control were conducted according to conventional management practices. All treatments used two pesticides, deltamethrin and bifenthrin, applied at rates of 15 g active ingredient·ha^−1^ and 12 g active ingredient·ha^−1^, respectively. Two applications were made during the growing season, with an interval of 10–15 days between applications, to reduce the risk of pest resistance.

Soil samples from the decomposition of in situ vegetable residue return were collected on 15 August 2023, 8 April 2024, and 29 August 2024. Soil samples from the 0–20 cm depth were collected using the five-point sampling method within each plot, with the five samples mixed to form one composite sample for each plot. Three composite samples were obtained per treatment (*n* = 3). The soil samples were passed through a 2 mm sieve, cleaned to remove impurities, and then placed in sealed bags, labeled, and stored in dry ice for transport back to the laboratory. Soil samples for ammonium nitrogen and nitrate nitrogen analysis were stored at 4 °C in a refrigerator, while samples for high-throughput microbial sequencing were stored at −80 °C. Other soil samples for different analyses were air-dried naturally [18].

### 4.3. Determination of Soil Physicochemical Properties

Soil physicochemical properties were determined according to standard methods: pH was measured using the potential method with deionized water (water-to-soil ratio 2.5:1.0); AK was determined using the NH_4_OAc extraction-flame photometry method; TN was measured using the Kjeldahl method; EC was measured according to HJ 802-2016 “Measurement of Soil Electrical Conductivity by Electrode Method”; AP was determined using the molybdenum-antimony colorimetric method; NH_4_^+^-N and NO_3_^−^-N contents were extracted using 2 mol/L KCl solution (soil-to-water ratio 1:5), followed by shaking and filtration, and the filtrate was analyzed using a continuous flow analyzer (AA3; Germany). The methods for soil parameter measurement followed the protocols described in Methods of Soil Agrochemical Analysis and Soil Agrochemical Analysis [44].

### 4.4. Determination of Soil Carbon Fractions

Easily oxidizable organic carbon (EOOC) in soil was determined using the KMnO_4_ oxidation method [45]. SOC was determined by the K_2_Cr_2_O_7_-concentrated H_2_SO_4_ external heating method [44]. Particulate organic carbon (POC) was measured by extraction with 5 g·L^−1^ sodium hexametaphosphate solution followed by potassium dichromate titration [46]. Mineralizable organic carbon (MOC) was determined using the closed incubation-alkali absorption method [47]. Dissolved organic carbon (DOC) was measured by the shaking extraction method followed by analysis on a Total Organic Carbon analyzer [48].

### 4.5. Determination of Soil Enzyme Activities

Soil enzyme activities were determined using enzyme activity kits from Beijing Solarbio Science & Technology Co., Ltd. (Beijing, China). Soil urease activity was measured using the indole phenol blue colorimetric method (BC0125) at a wavelength of 630 nm, with a standard curve drawn using NH_3_-N standard solution to calculate the NH_3_-N content in the samples. Enzyme activity was defined as the amount of enzyme that produces 1 μg NH_3_-N per gram of soil per day. β-glucosidase (S-β-GC) activity was measured using the p-nitrophenyl colorimetric method (BC0165) at a wavelength of 400 nm, with a standard curve established using p-nitrophenol standard solution. Enzyme activity was defined as the amount of enzyme that produces 1 μmol p-nitrophenol per gram of soil per day. Cellulase (S-CL) activity was measured using the 3,5-dinitrosalicylic acid (DNS) colorimetric method (BC0155) at a wavelength of 540 nm, with a standard curve established using glucose standard solution. Enzyme activity was defined as the amount of enzyme that produces 1 mg glucose per gram of soil per day. Peroxidase (S-POD) activity was measured using the o-phenylenediamine oxidation colorimetric method (BC0895) at a wavelength of 430 nm, with a standard curve established using a standard reagent. Enzyme activity was defined as the amount of enzyme that generates 1 mg of reaction product per gram of soil per day. Catalase (S-CAT) activity was measured using the ultraviolet spectrophotometric method (BC0105) at a wavelength of 240 nm, with enzyme activity defined as the amount of enzyme that catalyzes the decomposition of 1 mmol H_2_O_2_ per gram of soil per day.

### 4.6. Extraction and Sequencing of Soil Bacterial and Fungal DNA

Microbial community genomic DNA was extracted according to the instructions of the E.Z.N.A Soil DNA Kit (Omega Bio-tek, Inc., Norcross, GA, USA). The quality of the extracted genomic DNA was assessed using 1% agarose gel electrophoresis, and DNA concentration and purity were measured with a NanoDrop2000 (ThermoFisher Scientific, Inc., Waltham, MA, USA). Fungal community structure was analyzed using sequencing primers ITS (ITS1F-ITS2R): CTTGGTCATTTAGAGGAAGTAA and GCTGCGTTCTTCATCGATGC. The bacterial community structure was analyzed by amplifying the hypervariable regions V3-V4 of the 16S rRNA gene: 338F (ACTCCTACGGGAGGCAGCAG) and 806R (GGACTACHVGGGTWTCTAAT) [49]. The amplification program was as follows: 5 min pre-denaturation at 94 °C, followed by 30 cycles (denaturation at 94 °C for 30 s, annealing at 50 °C for 30 s, extension at 72 °C for 60 s), then a final extension at 72 °C for 7 min, and storage at 4 °C (ABI 9700 PCR System, Applied Biosystems, Inc., Waltham, MA, USA). The size of the amplified products was verified by 1% agarose gel electrophoresis at 170 V for 30 min. PCR products were purified using the Agencourt AMPure XP nucleic acid purification kit (Beckman Coulter, Inc., Brea, CA, USA). Library preparation was performed using the NEB Next Ultra II DNA Library Prep Kit (New England Biolabs, Inc., Ipswich, MA, USA), and sequencing was conducted on the Illumina MiSeq platform (Beijing Novogene Technology Co., Ltd., Beijing, China).

### 4.7. Bioinformatics Pipeline

Raw paired-end sequencing reads were first quality-filtered using fastp (version 0.19.6). Bases with a quality score lower than 20 at the read tails were trimmed using a 50 bp sliding window; when the average quality score within the window fell below 20, bases downstream of that window were removed. Reads shorter than 50 bp after trimming and reads containing ambiguous nucleotides (N) were discarded. The quality-filtered paired-end reads were subsequently merged using FLASH (version 1.2.11) based on overlapping regions between read pairs, with a minimum overlap length of 10 bp and a maximum mismatch ratio of 0.2 in the overlap region. Sequences not meeting these criteria were removed. The merged sequences were then demultiplexed according to barcode and primer sequences at both ends, and sequence orientation was adjusted accordingly. The optimized sequences were denoised using the DADA2 plugin implemented in QIIME2 (version 2020.2) with default parameters, generating amplicon sequence variants (ASVs) at single-nucleotide resolution based on error modeling. Sequences annotated as chloroplasts and mitochondria were removed from all samples prior to downstream analyses. To minimize the influence of sequencing depth on alpha and beta diversity analyses, samples were rarefied to 28,950 sequences per sample, corresponding to the minimum sequencing depth across all samples. After rarefaction, the average Good’s coverage was 99.90%, indicating a sufficient sequencing depth for microbial community analysis. Taxonomic annotation of ASVs was performed using a Naive Bayes classifier against the SILVA 16S rRNA database (v138) for bacteria and the UNITE database (v9.0) for fungi. The classification confidence threshold was set to 0.7.

### 4.8. Calculation of the Soil Quality Index (SQI)

To assess overall soil quality, a soil quality index (SQI) was calculated by integrating various soil biological, physical, and chemical properties. Principal component analysis (PCA) was used to construct a minimum dataset (MDS) of soil indicators [50], as detailed in Tables S2 and S3. The final minimal dataset selected includes NH_4_^+^-N, EOOC, POC, and S-CAT. Positive indicators (increasing values indicate better soil quality) included EOOC, particulate organic carbon (POC), and S-CAT, while the negative indicator (increasing values indicate poorer soil quality) was NH_4_^+^-N.

The SQI was calculated using the membership values and weights of the obtained soil indicators. The formula is generally structured as follows:

(1) $$SQI=\sum_{i=1}^{n}W_{i}\times S_{i}$$

where *n* is the number of indicators, and *S_i_* and *W_i_* are the score and weight of the *i*-th indicator, respectively. The larger the SQI value, the better the soil quality, with a linear relationship between the two.

### 4.9. Calculation of the Co-Occurrence Network

Co-occurrence networks were constructed separately for each treatment to explore potential associations among microbial taxa and environmental variables. Network analyses were performed at the genus level based on relative abundance data using R (version 3.3.1) and Python (version 2.7), with nine samples per treatment (*n* = 9). Prior to network construction, the top 50 genera ranked by relative abundance were retained, and unclassified taxa were excluded to reduce sparsity and low-abundance bias. In addition to microbial genera, selected environmental variables (e.g., soil pH and other measured physicochemical properties) were incorporated as nodes in the networks. Pairwise Spearman correlation coefficients were calculated among genera and environmental variables within each treatment. Only strong and statistically significant correlations (|*r*| ≥ 0.6 and *p* < 0.05) were retained to construct the networks. Network visualization and topological analyses were performed using Gephi (version 0.10.1). In the networks, nodes represent microbial genera or environmental variables, and edges represent significant correlations between nodes. Network topology metrics, including the number of nodes, number of edges, average degree, network density, and clustering coefficient, were calculated using Gephi’s Analyzer tool (version 0.10.1). Given that network topology parameters reflect structural complexity, these metrics were used to compare network complexity among treatments. Because correlations were calculated based on relative abundance data, the inferred relationships represent co-occurrence patterns rather than direct ecological interactions.

### 4.10. Statistical Analysis

The data were processed using Microsoft Excel 2019, and graphs were created with Origin 2024. One-way ANOVA and LSD multiple comparison were performed using IBM SPSS 25 to compare the differences in soil physicochemical properties, carbon components, enzyme activities, and microbial alpha diversity indices under different treatments (*p* < 0.05). The microbial community beta diversity differences between treatments were evaluated based on the Bray–Curtis distance algorithm, using NMDS and ANOSIM. The differences in microbial community composition between treatments were compared using the Kruskal–Wallis test and Dunn’s test, with *p* values adjusted using the FDR method. Data calculation and statistical analysis were performed using DPS 18.10. Path analysis was performed using the stepwise multiple regression module in DPS, following Wright’s path coefficient framework. Direct path coefficients were derived from standardized regression coefficients (Beta values), and indirect effects were calculated from the correlation matrix among predictors. Predictor variables were entered using a stepwise procedure. The sample size was *n* = 36. Model performance was evaluated using R^2^, F statistics, residual standard error, and the Durbin–Watson statistic. Multicollinearity was assessed using correlation matrices.

## 5. Conclusions

This study systematically explored the effects of in situ vegetable residue return on soil quality through a continuous three-season field experiment. The results demonstrated that in situ residue return is an effective strategy for improving soil fertility and quality. Soil nutrient analysis showed that in situ residue return improved soil physicochemical properties and carbon components. Specifically, HTJS increased SOC, TN, and MOC by 17.7%, 23.6%, and 43.4%, respectively. The activities of S-β-GC and S-POD in the HTJS treatment were significantly higher than those in other treatments after the third tillage. Analysis of microbial alpha diversity showed that the alpha diversity of bacterial and fungal communities was not significantly different among treatments, indicating that residue incorporation had no significant effect on soil microbial alpha diversity. Based on non-metric multidimensional scaling (NMDS) and differential species analysis, there were no significant differences in bacterial beta diversity among treatments, but in the HTJS treatment, the relative abundances of *Flavisolibacter* and *Devosia* were higher. Significant differences in fungal community beta diversity were observed among treatments. Although the relative abundance of Fusarium increased under HTJS, the field disease incidence index did not increase accordingly, indicating that microbial compositional shifts should be interpreted in the context of actual plant disease outcomes under continuous field cultivation. Co-occurrence network analysis revealed that the overall complexity of the fungal network was higher than that of the bacterial network. The SQI, constructed on the minimal dataset, showed that HTJS had the highest SQI (0.691) after the third tillage. Further path model analysis revealed that soil carbon components and the physicochemical environment were the main controlling factors influencing the SQI. In conclusion, after three consecutive seasons of residue return, the HTJS treatment was a simple and effective method that can optimize soil microbial communities, leading to a synergistic improvement in soil nutrients, carbon components, and overall soil quality, providing a sustainable utilization path for vegetable residues.

## Figures and Tables

**Table 1:** Soil quality index.

Treatments	Soil Quality Index		
	1st	2nd	3rd
CK	0.195	0.34	0.159
HTJ	0.401	0.617	0.505
HTJS	0.582	0.609	0.691
HTJN	0.493	0.651	0.677

## Data Availability

Data are contained within the article. The raw sequencing reads and associated metadata have been deposited in the NCBI Sequence Read Archive (SRA) under accession number SRP680007. The dataset is currently under embargo and will be publicly available upon publication.

## Supplementary Materials

The following supporting information can be downloaded at https://www.mdpi.com/article/10.3390/plants15071091/s1. References [51,52] are cited in the Supplementary Materials.

## Abbreviations

The following abbreviations are used in this manuscript:

CK	No residue return
HTJ	In situ residue return
HTJS	In situ residue return combined with a compound microbial inoculant
HTJN	In situ residue return combined with 100 kg/acre of ammonia solution
EC	Electrical conductivity
SOC	Soil organic carbon
TN	Total nitrogen
AK	Available potassium
AP	Available phosphorus
DOC	Dissolved organic carbon
POC	Particulate organic carbon
EOOC	Easily oxidizable organic carbon
MOC	Mineralizable organic carbon
S-UE	Soil urease
S-CL	Soil cellulase
S-CAT	Soil catalase
S-β-GC	Soil β-glucosidase
S-POD	Soil peroxidase
SQI	Soil quality index
MDS	Minimum dataset
NMDS	Non-metric multi-dimensional scaling
