# Supplementary material for: Response of Soil Quality and Microbial Community to In Situ Return of Vegetable Residues over Three Consecutive Cropping Seasons
Source: Plants (Basel). 2026 Apr 2;15(7):1091. doi: 10.3390/plants15071091 (PMC13074782; doi:10.3390/plants15071091)
Supplement: Supplementary file 1 [file plants-15-01091-s001.zip › plants-4097748-supplementary.pdf]

Response of soil quality and microbial community to in-situ return of vegetable residues over three consecutive cropping seasons

Haiying Wang [1,2,†], Zhizhuang An [1], Jianbin Liu [1], Liang Jin [1], Yan Li [3], Yu Hu [1], Jie Zhu [4], Mingjie Yao [1,5], Xuening Xu [1,2], Dan Wei [1,*], and Jianli Ding [1,*]

[1] Institute of Plant Nutrition, Resources and Environment, Beijing Academy of Agriculture and Forestry Sciences, Beijing 100097, China; why2814535432@163.com(H.W.); anzz@igsnrr.ac.cn(Z.A.); liujianbin@baafs.net.cn(J.L.); jinliang19762003@aliyun.com; huyu0805@126.com(Y.H.); yaomingjie1999@163.com(M.Y.); xvmm1316@163.com(X.X)
[2] College of Resources and Environment, Northeast Agricultural University, Harbin 150030, China
[3] Heilongjiang Academy of Black Soil Protection and Utilization, Harbin 150086, China; li.yan622@163.com
[4] College of Resources and Environment,Xinjiang Normal University, Wulumuqi 830054, China; zhujie190520@163.com
[5] College ofLight Industry Science and Engineering Tianjin University of Science and Technology, Tianjin 300457, China;
* Correspondence: wd2087@163.com(D.W.); lndjl@126.com (J.D.)
† These authors contributed equally to this work.

Supporting Information
Number of tables: 3
Number of figure: 1

***S1 Disease* assessment**

Field experiments were conducted to evaluate the occurrence and control efficacy of two major diseases of cabbage (Brassica oleracea var. capitata L.), namely soft rot and black spot.

Soft rot assessment

Soft rot was assessed at harvest (27 June 2024). All plants within each plot were visually inspected, and the number of diseased plants was recorded. Disease incidence (%) was calculated as the percentage of infected plants relative to the total number of plants per plot [1].

Black spot assessment

Black spot was evaluated at the peak disease stage (3 June 2024). Disease occurrence was investigated using a five-point sampling method in each plot. At each sampling point, four plants were examined, resulting in a total of 20 plants per plot. For each selected plant, one outer leaf was randomly chosen to determine disease incidence and severity.

Disease severity was assessed according to the grading criteria described by Zhang et al. [2], as follows:

Grade 0: No visible lesions.

Grade 1: Lesion area <1% of the leaf area.

Grade 3: Lesion area 2–5% of the leaf area.

Grade 5: Lesion area 6–10% of the leaf area.

Grade 7: Lesion area 11–20% of the leaf area.

Grade 9: Lesion area >20% of the leaf area.

Disease incidence (%), Disease index (%), and Control efficacy (%) were calculated using the following formulas：

$$\text{Disease incidence (\%)} = \frac{\text{Number of diseased plants}}{Total\ number\ of\ surveyed\ plants} \times 100 \qquad (1)$$

$$\text{Disease index (\%)} = \frac{\sum(\text{Number of diseased plants at each grade} \times \text{Corresponding grade value})}{\text{Total number of surveyed plants } \times 9} \times 100 \quad (2)$$

$$\text{Control efficacy (\%)} = \frac{\text{Disease index in control} - \text{Disease indexe in treatment}}{\text{Disease index in control}} \times 100 \quad (3)$$

Each treatment consisted of three replicated plots arranged in a randomized block design.

Table S1 Survey and control of diseases and pests

Treatment	Soft rot		Black spot			
	**Disease incidence (%)**	**Control efficacy (%)**	**Disease incidence (%)**	**Control efficacy (%)**	**Disease index (%)**	**Control efficacy (%)**
CK	9.31±1.27a	-	43.33±2.89a	-	14.44±0.96a	-
HTJ	5.85±1.1b	37.18±11.8a	36.67±2.89b	15.38±6.66b	4.07±0.32b	71.79±2.22b
HTJS	5.41±0.75b	41.88±8.08a	31.67±2.89bc	26.92±6.66ab	3.52±0.32bc	75.64±2.22ab
HTJN	4.6±0.05b	50.63±0.57a	26.67±2.89c	38.46±6.66a	2.96±0.32c	79.49±2.22a

Note: Data are presented as mean ± standard error (SE). Different lowercase letters within the same column indicate significant differences among treatments according to one-way ANOVA at $p < 0.05$.

S2 MDS Construction

Table S2 Principal component loadings and Norm values of soil indicators

Soil indicator	Principal component					Norm	Group	MDS
	PC1	PC2	PC3	PC4	PC5			
NH_4^+-N	-0.50	0.68	-0.30	-0.27	-0.09	1.49	2	Enter
NO_3^--N	-0.25	0.83	0.13	0.38	0.10	0.87	2	
pH	-0.60	-0.15	0.37	0.17	-0.36	1.72	1	
EC	0.15	-0.56	-0.08	0.03	0.76	0.94	2	
SOM	0.91	-0.04	-0.28	-0.01	-0.10	2.51	1	
TN	0.72	-0.08	-0.11	-0.40	0.27	2.04	1	
AP	0.39	-0.56	0.34	-0.20	-0.12	1.21	2	
AK	0.63	-0.32	-0.25	0.42	-0.22	1.83	1	
DOC	0.90	-0.12	-0.19	0.22	-0.18	2.48	1	
SOC	0.91	-0.04	-0.28	-0.01	-0.10	2.51	1	
EOOC	0.70	0.32	0.56	0.04	-0.06	1.96	3	Enter
POC	0.94	-0.04	-0.21	0.05	-0.14	2.59	1	Enter
MOC	0.81	0.37	0.42	0.02	-0.06	2.26	1	
S_CAT	-0.16	0.21	-0.34	0.67	0.32	0.99	4	Enter
S_CL	-0.42	-0.28	0.51	-0.16	0.01	1.23	3	
S_UE	0.03	0.50	-0.61	-0.48	-0.03	0.97	3	
S_β_GC	0.60	0.42	0.46	-0.07	0.42	1.79	1	
S_POD	0.82	0.44	0.34	-0.01	0.01	2.29	1	
Principal component eigenvalues	8.02	4.79	2.91	1.95	1.64			
Variance contribution rate/%	33.42	19.94	12.14	8.12	6.83			
Cumulative contribution rate/%	33.42	53.36	65.50	73.62	80.44			

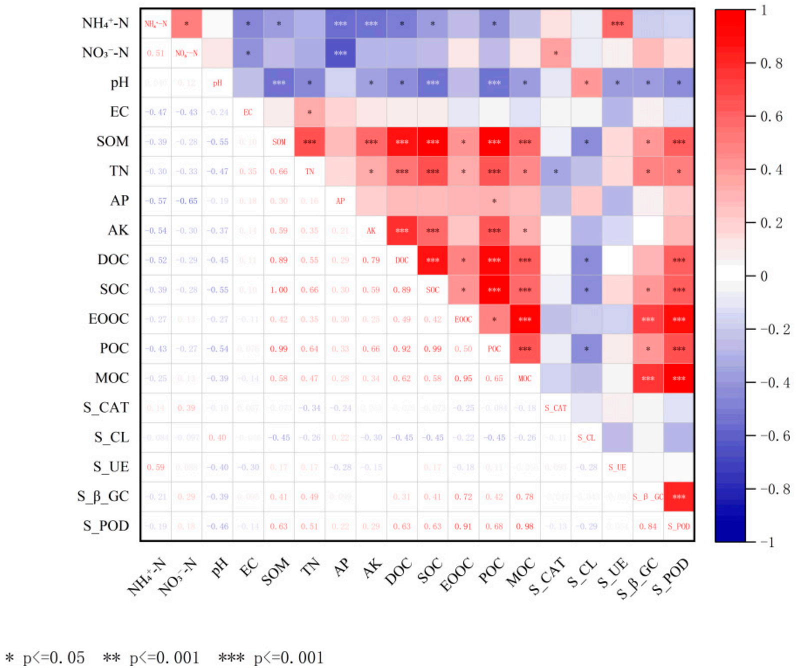

Figure S1 Pearson correlation analysis of soil quality evaluation indicators

Table S3 Common factor variance and weights for the Minimum Data Set (MDS) of soil quality evaluation

Soil indicator	MDS	
	Common Factor Variance	Weight
NH_4^+-N	0.476	0.082
EOOC	0.598	0.103
POC	0.652	0.112
S_CAT	0.163	0.028

Supplementary References

1. Zhang, Z.; Guan, C.; Xia, C.; Su, B. Screening of Chemicals for the Control of Black Spot in Chinese Cabbage. *Yunnan Agriculture* **2015**, 38–39.
2. Li, W.; Qin, J.; Jiao, J.; liu, Q.; Huang, Z.; Wu, X.; Chen, L.; Rong, R.; Wang, D.; Zhang, Z.; Yu, P. Effects of intercropping garlic combined with nongcanjing on control effect of soft rot, quality and yield of chinese cabbage. *Journal of Changjiang Vegetables* **2020**, 71–74.
